# The EU Green Bond Standard: A Plausible Response to the Deficiencies of the EU Green Bond Market?

**DOI:** 10.1007/s40804-023-00278-2

**Published:** 2023-03-13

**Authors:** Michal Pyka

**Affiliations:** Faculty of Law and Administration, Jagiellonian University, Cracow, Poland

**Keywords:** European green bonds, EU Green Bond Standard, EU Taxonomy, European Green Deal, Capital Markets Union, Market fragmentation

## Abstract

The article elaborates on the EU Green Bond Standard, a cornerstone of the Proposal for a Regulation on European green bonds that was put forward by the European Commission in July 2021. Within the analysis, three research questions are posed. First, the article deals with the question of whether the EU Green Bond Standard is really needed; particularly, whether there is a need for EU regulatory intervention on the EU green bond market. Second, it examines the method of regulation which was adopted for the EU Green Bond Standard. Specifically, does this standard differ and offer any added value compared to private standards for the issuance of green bonds? Finally, the article discusses the impact of the EU Green Bond Standard on the EU green bond market. Within this section, the questions of fragmentation and regulatory competition between public (EU) and private regimes are dealt with. The article ends with proposals for modifications of the EU Green Bond Standard. It has been suggested that the EU Green Bond Standard should have a mandatory character for all issuances of green bonds on the EU green bond market. Private standards should not be replaced by the EU Green Bond Standard but only reconciled with it. With regard to its content, the EU Green Bond Standard should constitute a mixture of public and private obligations; yet, some necessary regulatory space should be left for private standards.

## Introductory Remarks

In recent years, sustainability-themed investments in global capital markets have experienced an unprecedented growth.^1^ Among various sustainability-themed investment products, such as sustainability-linked loans or social bonds, green bonds occupy the principal place. The annual global green debt market reached $517.4 billion in 2021, which represents more than a 60% increase from $297 bn in 2020.^2^ These figures are expected to grow rapidly throughout the present decade to reach $1 trillion in 2023^3^ and $1.5 trillion in 2025.^4^ At the same time, the developed European green bond market accounted for $156 bn in 2021.^5^ In 2020, the euro became the main currency for green bond issuances, as almost 50% of the total global number of transactions were conducted in euros.^6^

The rapidly growing European green bond market has recently become one of the focal points of the European green revolution.^7^ In October 2021, the European Commission issued its first green bond, raising a total of €12 billion to be used exclusively to finance green and sustainable investments across the European Union (EU).^8^ As announced by EU Commissioner Johannes Hahn, the European Commission could issue up to €250 billion in green bonds as part of the post-COVID-19 recovery fund to help Member States conduct their environmentally friendly initiatives.^9^ Similarly, in the European Green Deal, the European Commission announced the establishment of a comprehensive framework for the transition to sustainability, targeting climate investments.^10^ The European Commission declared that it would establish the EU Green Bond Standard as an element of the European Green Deal.^11^

In July 2021, the European Commission published the Proposal for a Regulation on European green bonds (hereafter: Proposal for a Regulation on European green bonds, or the Proposal).^12^ The Proposal attempts to introduce the EU Green Bond Standard and thereby establish a comprehensive framework for the issuance of green bonds within the EU. Although at first glance the European Commission presented a convincing rationale for regulation of the EU green bond market, the details of the Proposal raise doubts as regards both the need for regulation and the method thereof. The aim of the present article is to elaborate on these issues and provide the reader with a precise answer to the question of whether the EU needs the EU Green Bond Standard and, if so, how such a standard should be shaped.

Specifically, the analysis should allow to find plausible answers to the following questions:

1. Is there a need for European regulation of the EU green bond market?
2. Which method of regulation will be adopted for the EU Green Bond Standard?
3. What will be the impact of the EU Green Bond Standard on the EU green bond market?

## Is There a Need for European Regulation of the EU Green Bond Market?

The Proposal for a Regulation on European green bonds serves both environmental and financial market purposes. Within the Proposal, both dimensions are interwoven with and equivalent to each other. The financial market dimension does not remain subsidiary to the environmental one, as may be assumed due to the strong connection between the Proposal and the European Green Deal.^13^ The Proposal remains part of the larger EU agenda on sustainable finance.^14^ It constitutes a first step towards the development of the comprehensive EU market for green bonds, thus contributing to the Capital Markets Union (CMU), as set in the CMU Action Plan of 2020.^15^ The primary objectives of the CMU are integration of national capital markets into a genuine European single market^16^ and overcoming fragmentation of the European financial market.^17^

The Proposal recognizes the likelihood that EU member states will adopt divergent measures and approaches regarding environmentally sustainable bonds;^18^ nonetheless, this observation does not remain the focal point of the Proposal. It appears that within the material scope of the Proposal the problem of fragmentation of national regimes has only a hypothetical character. The Proposal does not elaborate on this point. Instead, it implies that market fragmentation is caused rather by ‘the parallel development of market practices based on commercially driven priorities that produce divergent results’.^19^ Indeed, trade in green bonds remains largely unregulated—at both national and EU level. It is not only Europe-specific that the governance of green bonds remains decentralized and is primarily shaped by private governance regimes.^20^ The primary question which requires an answer in this context is whether this normative vacuum should be filled by legislative action undertaken at the EU level or not.

Careful examination of the Proposal leads to the conclusion that, for the European legislator, the systemic legitimacy context remains the center point of the reform of the EU green bond market. There is nothing surprising in this approach, since, as Chan remarked, for all markets in financial instruments, including the market in green bonds, the systemic legitimacy of the instrument’s purpose is paramount.^21^

To date, the EU green bond market remains not only self-made, but also largely self-regulating.^22^ Private regulation provided by private governance bodies, including the

International Capital Market Association (ICMA) and the Climate Bonds Initiative (CBI), is perceived as the market response to the absence of public regulation,^23^ while public regulation is in its infancy.^24^ Private governance bodies provide legitimacy on the green bond market through validation (certification) frameworks^25^ based on their own standards, including the Green Bond Principles (GBP)^26^ and Climate Bonds Standard (CBS).^27^

Deficiencies of private governance and self-regulation of capital markets are commonly known, especially after the 2008 financial crisis. In recent years, the space for self-regulation has been considerably narrowed. Within the EU, the most notable example is the market for EU rating agencies, which has become the subject of comprehensive regulation at the EU level.^28^ In this context it is not surprising that the EU green bond market is also expected to follow this path and open itself up to public regulation.

Self-regulation of capital markets can result in regulatory pluralism,^29^ which, as such, is not a negative phenomenon but can sometimes lead to fragmentation and dispersion of the legal order,^30^ as well as raise transaction costs and legal uncertainty.^31^ Yet, the picture of the EU green bond market is not unequivocally negative but more nuanced. The lack of a uniform legal standard^32^ for the issuance of green bonds appears to exert twofold effects on the EU green bond market. On the one hand, private regulation results in regulatory competition between private governance regimes and regulatory arbitrage applied by market participants,^33^ which can lead to ‘the race to the bottom’ phenomenon.^34^ A race to the bottom can increase the risk of greenwashing,^35^ which is often identified as a major risk facing green bonds.^36^ As a result of regulatory competition, private regulatory regimes would not provide any harmonious approach, which would result in regulatory uncertainty on the EU green bond market.

On the other hand, regulatory competition inherent in private regulation does not necessarily result in a race to the bottom. Pluralistic, diversified and constantly changing approaches adopted by private governance regimes enable them to adapt to the expectations of market participants^37^ and, therefore, to perform functions that warrant the continuous development of the green bond market, including maintenance of the high level of confidence of investors in the economic value and environmental impact of green bonds.^38^ As a consequence, private regulation of the EU green bond market should not necessarily result in a lowering of standards of green bonds issuance.

Currently, there are no visible signs of a race to the bottom on the EU green bond market which could justify regulatory intervention at the EU level. Market fragmentation as such should not be perceived as a legitimacy concern as long as regulatory competition between private governance regimes does not result in a race to the bottom and a lowering of standards of green bonds issuance.

## What Method of Regulation Was Adopted for the EU Green Bond Standard?

### Method of Regulation of the EU Green Bond Standard—Comparison with Private Regulation

This section of the article will discuss what method of regulation was adopted for the EU Green Bond Standard. Will this standard function similarly to private standards for the issuance of green bonds? To answer this question, which is of fundamental importance for the future shape of the EU green bond market, a wider context of the CMU architecture should be provided first.

As pointed out in Sect. 2, compared with other capital markets, the EU green bond market is still in its infancy. This, and not any decision of the EU or its member states, is the reason why this market is regulated predominantly by private regulatory bodies. In principle, the present architecture of the EU green bond market remains in accordance with the CMU objectives. Since the CMU was designed as a public-private governance regime,^39^ it supports the establishment of hybrid public-private regulatory frameworks.^40^ Within the CMU, the regulatory space remains open for private regulators that provide the EU capital markets with many non-coercive compliance mechanisms, such as contractual standards for the issuance of financial instruments, codes of conduct, and comply-or-explain mechanisms.^41^

The Proposal for a Regulation on European green bonds serves as a game changer for the EU green bond market, because it takes the form of a directly applicable EU regulation, aiming to achieve maximum harmonization^42^ and diminish the space for private regulation.^43^ It remains in line with the development of institutional governance for EU capital markets, which is subject to an incremental evolution towards an intensification of supervisory governance at the EU level.^44^ In recent years, the ‘top-down’ approach to building the CMU architecture has become the dominant method of regulation.^45^ Specific markets in financial instruments are reinforced against fragmentation risks.^46^ In overcoming fragmentation, they become more dependent on the interaction between the EU and national regulatory regimes^47^ than on private governance. This development remains in line with the CMU objective, which is to establish an overreaching financial regulatory framework within the EU capital markets.^48^

The Proposal for a Regulation on European green bonds fits the trend of building the CMU architecture using the ‘top-down’ approach. In fact, it constitutes a representative example of the regulatory approach that is often referred to as European Regulatory Private Law.^49^ Within this approach, private law is used for regulatory purposes at the EU level.^50^ Private law is aimed not at achieving corrective justice between market participants but at reaching specific policy goals on the market agenda.^51^ A perfect example of this approach is the EU law that governs the relations between customers and investment firms in the EU retail financial markets.^52^

Since European Regulatory Private Law serves primarily specific public policy goals, it remains ill-suited to perform its primary private law function, namely to resolve conflicts between private parties. It relies on administrative enforcement by regulatory agencies that are designed specifically to ensure the functioning of the respective markets rather than on judicial enforcement by individual private parties.^53^ The deprivation of the primary function of private law is additionally increased when private law remedies are accompanied by public sanctions with respect to the same obligations.^54^

It is already clear from the Explanatory Memorandum to the Proposal for a Regulation on European green bonds that the EU Green Bond Standard is aimed at realizing public purposes, namely those of the European Green Deal (environmental) and those of the CMU (financial market). One of the major features of the EU Green Bond Standard is the practical abandonment of private enforcement. The EU Green Bond Standard will be based on four key requirements, all of which involve the use of public (administrative) enforcement mechanisms.^55^ Detailed regulation of European green bonds set forth in the Proposal is based on public control over the issuance of these bonds with the use of sanctions which are administrative in their nature. Does this approach differ from the private regulation provided for the EU green bond market by private regulatory bodies?

It would be a misunderstanding to maintain that private regulation on the EU green bond market is centered on the introduction of mechanisms of private enforcement of particular obligations stemming from the terms and conditions of green bonds. In reality, private standards for the issuance of green bonds are not focused on private enforcement. Instead, they introduce several quasi-administrative enforcement mechanisms, including reporting obligations in relation to the use of proceeds from green bonds.^56^ The only substantial difference between private regulation and the EU Green Bond Standard is the intensity of the EU normative framework for European green bonds, under which administrative supervision over the issuances of European green bonds is more far-reaching than in the case of private standards.

At first glance, the described method of regulation of the EU Green Bond Standard appears to adequately supplement current market regulation provided for by private regulatory bodies. Since the EU Green Bond Standard has been envisaged as a further step in the development of the EU green bond market and as a standard which, at least for some time, will complement rather than replace private standards for the issuance of green bonds,^57^ it seems natural that it should be shaped parallel to these standards. Unfortunately, what may be viewed as a major advantage of the EU regulation, in reality constitutes its major drawback and a serious impediment for the future growth of the EU green bond market. Since the EU Green Bond Standard will be constructed in parallel to private standards, it will not constitute a plausible response to the deficiencies of private regulation. It will focus on administrative enforcement and not adopt any mechanisms of private enforcement that could help stabilize relations between issuers and holders of European green bonds. Regrettably, it will not interfere with the terms and conditions of European green bonds. Specifically, it will not introduce any obligation to incorporate into the terms and conditions any provisions on the use of proceeds from bonds and/or reporting mechanisms^58^ that could be enforceable by holders of European green bonds.

The above deficiencies of the EU Green Bond Standard make it appropriate to consider the question of protection of bondholders against the so-called ‘green defaults’, the field of regulation where the need for effective private enforcement mechanisms remains the most urgent.

### Protection Against Non-Performance of ‘Green’ Obligations (‘Green Defaults’) Under the EU Green Bond Standard – Lack of Private Enforcement Mechanisms?

Green bonds are generally ill-suited to protect their holders against ‘green defaults’, that is, against events in which an issuer of a green bond does not meet its environmental commitments towards bondholders.^59^ ‘Green default’, which is often a consequence of greenwashing, usually occurs when proceeds from a green bond are not applied to a green project or when a green project loses its ‘green’ characteristic before the maturity date of a green bond.^60^ Green default events encompass not only deliberate mis-selling of ‘brown green’ bonds by issuers, but often also an insufficient progress in fulfilling environmental obligations incurred by the issuers of truly ‘green’ bonds.

The contractual relationship between an issuer of a green bond and a bondholder usually does not equip the latter with any tools in case of a green default.^61^ Green bonds generally do not endow bondholders with any direct influence on the performance of issuers,^62^ including the management of green projects.^63^ Private standards for the issuance of green bonds sometimes require reporting of non-conformance with environmental commitments of the issuers, which, if not remedied, can result in loss of certification.^64^ Further consequences have been left to contractual stipulation, which means that they are uncertain.^65^ Environmental goals are insufficiently mirrored in private law obligations incurred by the issuers of green bonds. Green bond interest rates are generally not related to the performance of the issuer in managing green projects.^66^ To make this picture more nuanced, green bond documentation sometimes includes the express reservation that non-fulfilment by the issuer of the environmental obligations will not constitute a contractual event of default.^67^ It would not be an overstatement to say that green bonds are generally structured in a way that mirrors conventional bonds and that they are ‘green’ only by name and not by their provisions.^68^ The reason why green bond provisions are generally silent on green default issues lies in the fact that the terms and conditions of green bonds are shaped to serve primarily the interest of issuers. Bondholders accept ill-suited terms and conditions for green bonds, because they do not have enough time and resources to engage in a process of bond restructuring.^69^

According to Chan, lack of contractual stipulations on green default events constitutes a major legal challenge facing green bonds today.^70^ In the absence of specific contractual provisions on green default, the risk of greenwashing remains perilously high.^71^ Reputational risks for issuers engaging in mis-selling of bonds labelled ‘green’ despite having no plans of significant involvement in green projects remain insufficient to prevent greenwashing and provide an adequate level of legal security on the green market.^72^ In cases of green default, bondholders are basically left with the option of quitting their investments in green bonds.^73^ Yet, an exit strategy does not appear to be a plausible solution for bondholders because the demand for defaulting green bonds on the secondary market usually remains relatively low.

The Proposal for a Regulation on European green bonds does not sufficiently address the question of green defaults. The Proposal introduces several authorizing, monitoring and reporting obligations. Particular emphasis has been placed on public control over the management of the underlying green projects, including their ongoing evaluation linked to administrative sanctions. Unfortunately, the mechanisms of public enforcement of these obligations turn out to be ill-suited to provide the EU green bond market with an adequate level of protection against the risk of green defaults. Some of the deficiencies of private regulation provided for by ICMA and CBI have been repeated. For example, the Proposal does not set a procedure for withdrawing the EU ‘green’ label^74^ of green bonds, other than the possibility of including a statement in the post-issuance review that the bond does not meet the EU Taxonomy requirements.^75^ It resembles the GBP and CBS standards stipulating the loss of certification of green bonds in case of green defaults.

The legislative content of the Proposal is based on the assumption that the deficiencies of private regulation of the EU green bond market, which keep the risk of green defaults on a perilously high level, can be remedied by way of increased public control over the issuance of European green bonds. This approach seems to neglect the possible benefits of private enforcement mechanisms and remains surprising in light of the CMU objectives. Since the CMU is expected to create a hybrid, public-private regime for trade in financial instruments, where private enforcement mechanisms play an essential regulatory role, the role for private enforcement in the EU Regulation on European green bonds should be warranted. In fact, public enforcement of the obligations of European green bond issuers may prove insufficient to achieve both financial markets’ and environmental objectives. Despite positive opinions of external reviewers of European green bonds, particular bondholders could be dissatisfied with the performance of the underlying green projects. They should be entitled not only to vote with their feet and sell out their bonds, which will result in a fall of their prices, but also to apply contractual remedies, such as increased coupon rates. Under the present shape of the Proposal, after public authorities grant European green bonds a ‘license’ to operate, which constitutes a proof of legitimacy of these instruments, bondholders are generally deprived of effective private enforcement mechanisms regarding these bonds.

Regrettably, neither the Proposal nor the amendments thereto that were put forward by European Parliament Rapporteur Tang in December 2021 and adopted by the Committee on Economic and Monetary Affairs of the European Parliament in May 2022^76^ touch upon the question of private enforcement of the authorizing, monitoring and reporting obligations borne by the issuers of European green bonds. The Proposal does not require from the issuers of European green bonds that they include in the issuance documents any private law sanctions in case of a green default. It would be plausible for the EU Green Bond Standard to effectively tackle the question of green defaults within the sphere of private law. The EU Green Bond Standard should require specific obligations of a private law nature to be incorporated into terms and conditions of European green bonds in order to attach the EU label to them. Remedies for green defaults could include the right of bondholders to demand accelerated repayment or to receive an increased coupon to be paid by the issuer (a ‘margin ratchet’ remedy).^77^ Such an approach by the European legislator would substantially narrow the space for European green bonds; nevertheless, it would create a strong incentive for issuers to achieve environmental goals, which is one of the primary purposes of the Green Bond Standard.^78^ Simultaneously, it would stabilize relationships between issuers and holders of European green bonds, thus contributing to the achievement of the CMU objectives. Finally, it would constitute a strong incentive for the assimilation of green bonds and green loans. Currently, it is the green loans that have enforceable green covenants more frequently.^79^ Prominent examples of these covenants are clauses on margin ratchet remedies that have been successfully incorporated into the contractual stipulations of green loans.^80^

Under the current shape of the Proposal for a Regulation on European green bonds, private enforcement mechanisms have been left to contractual stipulations incorporated into the terms and conditions of European green bonds. As in the case of infringements of other capital market rules, private law enforcement should therefore be governed by divergent national regimes.^81^ Bearing in mind the specific nature of green bonds, under which bondholders do not suffer any material loss of an economic nature but, instead, losses generated as an effect of green defaults are of an environmental character, application to these losses of private law remedies recognized by national laws can prove impossible.

### First Partial Conclusion

The specific method of regulation of the EU Green Bond Standard, entailing a wide array of obligations imposed on issuers accompanied by public (administrative) enforcement mechanisms, will probably enable European green bonds to efficiently perform their public (environmental) functions. Additionally, this method of regulation remains in accordance with the CMU objectives. Regrettably, the domination by public obligations and public enforcement mechanisms means that the EU Green Bond Standard copies major deficiencies of private standards for the issuance of green bonds, especially the weak position of bondholders. Admittedly, European green bonds issued under the EU Green Bond Standard will provide bondholders with an enhanced level of protection against green defaults as compared with protection provided by green bonds issued under private standards for the issuance of green bonds. However, the enhanced level of protection against green defaults will be achieved only thanks to the use of public (administrative) enforcement mechanisms. European green bonds will not constitute any substantial added value for bondholders as regards their contractual position towards issuers, since they will not equip them with any mechanisms of private enforcement of the obligations incurred by the issuers. Although the incorporation of such mechanisms into the EU Green Bond Standard would be onerous to issuers, they would be rewarded by growing market demand from environmentally aware investors, who are increasingly focused on green and sustainable investments.^82^ The cost of making European green bonds more open to private law will definitely be worth the effort. To date, it seems like European green bonds are constructed more like public law instruments than private law instruments.

## What Will Be the Impact of the EU Green Bond Standard on the EU Green Bond Market?

### The EU Green Bond Standard as a Remedy for Market Fragmentation?

The Proposal for a Regulation on European green bonds has been envisaged as a remedy for fragmentation of the EU green bond market, which was generated by regulatory pluralism of private governance regimes. As has been discussed in Sect. 1, market fragmentation as such does not constitute a legitimacy concern that should be addressed at EU level. From this perspective, the EU Green Bond Standard does not appear suitable for its purpose. This section of the article will assess whether this standard will exert any impact on market fragmentation.

To begin with, it is worth emphasizing that since the EU Green Bond Standard has been projected to complement rather than replace private standards,^83^ it remains doubtful whether it will bring ‘clarity, transparency, and coherence’ to the EU green bond market, as envisaged by the European Commission.^84^ As the Proposal should be perceived in the wider context of the EU market for sustainable investments, its material scope proves too narrow to overcome the existing market fragmentation. It covers only green bonds and not all GSS (‘green’, ‘social’ and ‘sustainable’) bonds.^85^ Differences between particular markets in GSS bonds do not justify partial regulation that covers only the green bond market. The amendments to the Proposal that were put forward in the Report of the Committee on Economic and Monetary Affairs of the European Parliament of May 2022 better reflect the specific nature of GSS bonds, in which various elements (‘green’, ‘social’ and ‘sustainable’) are frequently combined within one legal instrument (bond). In Article 63a of the amended version of the Proposal for a Regulation on European green bonds, the Committee proposes that, as part of an evaluation report, it should be assessed on a five-year basis whether the scope of the Regulation on European green bonds should be extended to include bonds whose proceeds are allocated to an economic activity that contributes to a social objective.^86^

To sum up, the Proposal for a Regulation on European green bonds provides for only partial regulation of the EU markets in GSS bonds, since it applies only to the EU green bond market. As a result of its narrow scope of application, the projected EU Green Bond Standard will not diminish but rather increase market fragmentation. In the future, after the adoption of EU measures aimed at regulation of the EU markets in social and sustainable bonds, market fragmentation will further increase, since the EU markets in GSS bonds will be governed by three potentially conflicting sets of regulations.

### Facilitation of Competition Between the EU and Private Governance Regimes

It appears that the EU Green Bond Standard has been projected to diminish regulatory space for private standards for the issuance of green bonds and to eventually displace them. To date, the fact that the EU Green Bond Standard will not instantaneously replace the existing private standards will lead to regulatory competition between the public (EU) and private governance regimes and to diminishment of market space for the latter. For this reason, the question of the character of relations between the EU Green Bond Standard and private standards deserves elaboration, which will be provided in this section of the article.

Even before the adoption of the Proposal for a Regulation on European green bonds, fragmentation of the EU green bond market was noted to facilitate competition not only between private governance regimes, but also between these regimes and public regulation.^87^ For this reason it is not surprising that if the EU Green Bond Standard is introduced, market space for private governance regimes will be diminished as a result of competition between public (EU) and private regimes. Consequently, the environmental impact provided by the EU green bond market will be impaired.

In the short term, even if, eventually, the EU Green Bond Standard appears to perform its environmental functions better than private regulation, increased competition between private and public governance regimes could deter investors from investing in the EU green bond market. In the long term, the EU Green Bond Standard has the potential to eliminate private regulation from the EU green bond market as it offers incentives that are absent within private regulation. Private regulation will be disincentivized^88^ and deprived of its competitive advantages by public (EU) regulation. Even without these incentives, the EU will be better positioned to enter into competition with private governance regimes, simply because of the fact that the EU will play a dual role: a quasi-private regulator within the sphere of the EU Green Bond Standard and a public regulator within the sphere of supervision over private regulatory bodies. The boundaries between those two spheres will be blurred to a significant extent, which allows the prediction that the EU will occupy a dominant position in a competition with private governance regimes. In light of the above, the emergence and functioning of the European Green Bond Standard will exert negative effects on the EU green bond market, both from environmental and financial market perspectives.

### The Modes of Competition Between the EU and Private Governance Regimes

In the green bond market it is generally recognized that green bonds can be classified as belonging to different categories, depending on different ‘shades of green’^89^ of the underlying green projects, namely ‘dark green’, ‘medium green’ or ‘light green’. Green bonds aimed at financing green projects that bring the most significant environmental benefits are named ‘dark green’ bonds as opposed to ‘medium green’ and ‘light green’ bonds. At first glance, European green bonds seem to be designed to play the role of ‘dark green’ bonds. Not surprisingly, however, the ambitions of the EU go further. The EU Green Bond Standard which the EU seeks to establish and promote will be higher than all the ‘green shades’ standards currently recognized on the green bond market.^90^ As remarked by the European Commission:

Once it is adopted by co-legislators, this proposed Regulation will set a gold standard for how companies and public authorities can use green bonds to raise funds on capital markets to finance such ambitious large-scale investments, while meeting tough sustainability requirements and protecting investors.^91^

These elevated expectations arise from the fact that the EU Green Bond Standard should introduce objectively verifiable and transparent requirements for qualification as a European green bond,^92^ which will be based on the EU Taxonomy.

By applying and promoting the EU Green Bond Standard as a special ‘gold’ standard, the EU enters into regulatory competition with private regulatory bodies. Not only will the EU Green Bond Standard considerably narrow the regulatory space for ICMA and CBI, but it will also bring a great deal of regulatory ‘chaos’ to the EU green bond market. European green bonds will be positioned on this market as more ‘environmentally friendly’ instruments than green bonds issued under the auspices of ICMA and CBI and labelled as ‘gold’ bonds. It could lead to elimination from the EU green bond market of green bonds other than the European ones and to a regulatory monopoly of the EU on this market.^93^ However, the recent Opinion of the European Central Bank (ECB) leaves no doubt that from the EU perspective, the EU Green Bond Standard should be shaped, if not as a mandatory standard, then as the primary green bond standard within the EU.^94^ It stems from the ECB Opinion that the desired outcome of the regulatory competition between the EU and private regimes should be a dominant position or even monopoly of the EU regime. The EU regime is designed to gradually diminish the space for private regulation, which is used by the EU legislator as a tool in the transformation of the EU green bond market from private to public regulation and not as a creator of legal norms applicable within a public-private, hybrid regulatory framework,^95^ which could remain in line with the CMU objectives.

Considerable doubts are also raised about the character of the EU Green Bond Standard as the ‘gold’ standard applicable to European green bonds. Does it mean that European green bonds will be designed only for the purposes of financing ‘extraordinary’ green projects that will bring environmental benefits which exceed the benefits provided by currently known ‘dark green’ bonds? This would exert negative effects on the environmental purposes of the European Green Deal, because a lower number of green projects would be labelled ‘green’ at the EU level and receive financing from European green bonds.^96^

If a much wider scope of application of the EU Green Bond Standard were adopted, which seems more probable on the grounds of the projected wide scope of application of the EU Taxonomy,^97^ it would mean that all green projects financed by European green bonds would receive the one EU ‘green’ label. Since the EU Green Bond Standard – in the opinion of the European Commission cited above – should be perceived as a ‘gold’ standard, all European green bonds would function as de facto ‘dark green’ bonds. This would undermine the status of a ‘dark green’ bond and send an ambiguous signal to market participants. If European de facto ‘dark green’ bonds did not provide extra environmental benefits compared to their non-European counterparts, they would nonetheless gain financing on the EU green bond market on the sole basis of their EU ‘green’ label, while non-European green bonds would face worse conditions for such financing, even despite being classified as ‘dark green’ bonds. Most likely, for green bonds issued under the ICMA or CBI standards it would be much more difficult to receive the status of ‘dark green’ bonds on the EU green bond market due to the lacking EU ‘green’ label. Mitigation of the risk of uncertainty on the EU green bond market would be difficult, since no clear rating system that would enable differentiation between distinct types of European green bonds was introduced by the European legislator. Instead, informal ratings, with the use of several ‘green shades’ standards, would probably be promoted by external, non-EU auditors, certifying agencies, etc. It would result in a great deal of regulatory uncertainty on the green bond market.

### Second Partial Conclusion

The EU Green Bond Standard does not appear as a plausible solution for fragmentation of the EU green bond market – simply because of the fact that market fragmentation does not constitute a legitimacy concern that should be addressed. Contrary to its purpose, the EU Green Bond Standard will not reduce but rather increase market fragmentation. This will happen not only due to the narrow scope of application of this standard, which does not cover bonds other than ‘green’ GSS bonds. An increase in market fragmentation will occur mainly because of the fact that the EU Green Bond Standard was projected to function as a standard complementary to the existing private standards for the issuance of green bonds. The role of the EU Green Bond Standard is ambiguous, to say the least. Undoubtedly, it will facilitate competition between public (EU) and private governance regimes, which will lead to private standards being deprived of their competitive advantages and, in the long term, to elimination of these standards from the EU green bond market. The EU Green Bond Standard will play the role of ‘gold’ standard, which will probably mean that European green bonds, due to their EU ‘green’ label, will be perceived as ‘dark green’ bonds by market participants. All of these developments will bring about a great deal of regulatory uncertainty on the EU green bond market.

## Concluding Remarks

The answer to the question posed in the title of this article is negative. The EU Green Bond Standard cannot constitute a plausible response to the deficiencies of the EU green bond market, primarily due to the false assumptions upon which this standard was built. Contrary to what was stated in the Proposal for a Regulation on European green bonds, deficiencies in the EU green bond market do not entail market fragmentation, but rather a lack of effective private enforcement mechanisms, especially those that could protect bondholders against ‘green defaults’. This is the main reason why green bonds are ill-suited to fulfil their environmental and financial market functions. The EU Green Bond Standard will do more harm than good to the EU green bond market, since it does not introduce any effective private enforcement mechanisms and, even more importantly, disrupts modes of competition between various standards for the issuance of green bonds. To address the shortcomings of the EU Green Bond Standard in the shape it is presented in the Proposal for a Regulation on European green bonds, the following modifications to this standard could be worth considering:

1. The EU Green Bond Standard should not function as a complementary but as an exclusive standard for all green bond issuances on the EU green bond market, including those based on private standards for the issuance of green bonds. All green bonds issued on the EU green bond market, including bonds issued on the basis of private standards for the issuance of green bonds, should be referred to as ‘European green bonds’.
2. Private standards for the issuance of green bonds should be reconciled with the EU Green Bond Standard in order to be applicable on the EU green bond market. Reconciliation should entail mandatory alignment of green bonds to the EU Taxonomy, as well as introduction of external review obligations, and supervision by ESMA and national supervisory authorities over green bond issuances. There should be no differences between European green bonds with respect to these public obligations.
3. Private standards for the issuance of green bonds reconciled with the EU Green Bond Standard should introduce effective private enforcement mechanisms for green bond obligations incurred by issuers and holders of European green bonds, including protection against ‘green defaults’. Besides, they should be entitled to introduce additional private law obligations.
4. The scope of application of the EU Green Bond Standard should be extended to the issuance of social bonds and sustainable bonds.

The above modifications to the EU Green Bond Standard should address deficiencies of the EU green bond market which result in insufficient fulfilment of financial market and environmental functions by green bonds. They should contribute to the creation of a comprehensive framework for the EU green bond market without detracting from its public-private character based on regulatory competition between private governance regimes that remain subject to public control.

## Footnotes

1 UNCTAD (2021).

2 Jones (2022).

3 Liboreiro (2021).

4 Ibid.

5 Statista Research Department (2022).

6 Liboreiro (2021).

7 Park (2018), p 4.

8 Sivaramakrishnan et al. (2021).

9 Liboreiro (2021).

10 European Commission (2020a), p 1: ‘Establish an EU Green Bond Standard, in 2020 and explore how this standard, as well as other enabling frameworks, can increase public and private finance for sustainable investments’.

11 Ibid., p 11.

12 European Commission (2021a).

13 Recital 2 to the Proposal for a Regulation on European green bonds.

14 Explanatory Memorandum to the Proposal for a Regulation on European green bonds, p 1. Also, European Commission (2018).

15 European Commission (2020b).

16 Ibid., p 12.

17 Ibid. Also Valiante (2018), p 23, who emphasizes that the EU has ‘experienced significant financial fragmentation in recent years, while capital markets rely on a complex interaction between national and EU regulatory regimes’.

18 Recital 5 to the Proposal for a Regulation on European green bonds. Also, Explanatory Memorandum to the Proposal for a Regulation on European green bonds, p 5.

19 Recital 5 to the Proposal for a Regulation on European green bonds.

20 Park (2018), p 1.

21 Chan (2021), p 224.

22 Ibid., p 230.

23 Banahan (2019), p 848.

24 Park (2018), p 1: ‘Largely missing from the still-nascent green bond market are government regulators’.

25 Ibid., p 35: ‘… the greenness of a green bond is determined by a multi-modal validation framework governed by private third-party organizations that safeguard the market from the risk of greenwashing’.

26 International Capital Market Association (2021).

27 Climate Bonds Initiative (2017).

28 Banahan (2019), p 848: ‘In the past, the market has sought to address the vacuum left by regulatory agencies without success. The most notable example of market self-regulation comes from the 2008 Financial Crisis, where credit agencies, playing a similar role to green bond second opinion verifiers, failed to give ratings adequately reflecting the investment risk.’

29 As noticed by Senn (2011), p 98, self-regulatory associations and business enterprises add to legal pluralism.

30 Ibid.

31 See Rödl (2008), p 746, who notices that private law pluralism raises transaction costs and legal uncertainty.

32 Freeburn and Ramsay (2020), pp 436–437.

33 Park (2018), p 31: ‘A second type of governance challenge is regulatory arbitrage. The existence of multiple private governance regimes, … allows a firm to select a regulatory framework that is most conducive to its interests’. In case of green instruments, market participants can exploit gaps between private CSR standards to engage in greenwashing. See ibid.

34 Private governance regimes provide for insufficient enforcement measures, when compared with public regimes: Chan (2021), p 231; Freeman (2000), pp 824–825.

35 Note the definition of greenwashing proposed by Ruiz-Blanco et al. (2022), p 4025, according to whom greenwashing should be described as ‘… the difference between what the company says it does in terms of commitment to sustainability, and what the company actually does as evaluated by external parties’. On the risk of greenwashing as an effect of regulatory pluralism on the green bond market: European Central Bank (2021), pt. 1.7: ‘Effective international cooperation … will be essential to ensuring the international coherence of standards for green bonds and avoiding the risk of competitive races to the bottom, which could exacerbate the risk of greenwashing and globally undermine the positive environmental impact of green bonds.’

36 Talbot (2017), p 129.

37 Park (2018), p 30: ‘Procedurally, private governance is often faster to implement and arguably more responsive to the needs of market participants’.

38 As rightly pointed out by Park (2018), p 34: ‘The future of the green bond market hinges on the capacity of issuers to sell more green bonds and the confidence of investors and civil society in their economic value and environmental impact’. The confidence of investors is warranted mainly by the high quality of reviews of green bonds provided by green bonds verifiers: Rose (2018), p 59.

39 Dorn (2016), p 85.

40 Park (2018), p 44.

41 Ibid.

42 Explanatory Memorandum to the Proposal for a Regulation on European green bonds, p 6.

43 More on this issue, see Sect. 4.2 of this article.

44 Moloney (2016), pp 411–412.

45 Langenbucher (2020), pp 601–603; Constâncio et al. (2019), p 4.

46 Moloney (2016), p 395.

47 Valiante (2018), p 23.

48 CMU Action Plan, p 14: ‘Gradual progress towards more integrated capital markets supervision will be indispensable’.

49 Comparato (2016), pp 621–626. The term European Regulatory Private Law was first used by Micklitz (2009).

50 Wallinga (2020), p 5; Marcacci (2017), pp 305–332.

51 Wallinga (2020), p 5. Also, Collins (2018), p 321: ‘Whilst traditional private law may embrace instrumental goals such as protection of the weaker party, the distinguishing characteristic of techno-law is its focus on a single goal—the promotion of the single market—at the expense of all others. Furthermore, the dimension of traditional private law systems of balancing the interests and rights of the parties to the transaction by providing a system of corrective remedies typically plays a subsidiary role in the EU directives, and in some it may be omitted altogether.’

52 Cherednychenko (2021), p 154.

53 Micklitz (2011), pp 564–567.

54 Tountopoulos (2019), p 723.

55 These are: EU Taxonomy alignment, which means that the funds raised by the bond should be allocated fully to projects that are aligned with the EU Taxonomy; full transparency on how the bond proceeds are allocated through detailed reporting requirements; external review, under which European green bonds will be checked by external reviewers to ensure compliance with the EU Regulation and alignment of the funded projects with the EU Taxonomy; and supervision over external reviewers: European Commission (2020c).

56 International Capital Market Association (2021), p 6.

57 The EU Green Bond Standard has been designed as a voluntary standard: European Commission (2021b), p 4: ‘This proposal will create a high-quality voluntary standard, available to all issuers to help attract sustainable investments’.

58 Doran and Tanner (2019), p 26.

59 Freeburn and Ramsay (2020), p 440.

60 Corke et al. (2019).

61 Freeburn and Ramsay (2020), p 440: ‘Generally, it appears that the terms of many green bonds may be either silent or expressly exclude rights of investors in the event of a default. Typically, the contractual documentation associated with a green bond will not support any claims against an issuer in the case of a green default’; Chan (2021), p 249: ‘The events of default clauses and covenant clauses are typically confined to details of payment performance and are silent on the issuer’s obligations of reporting and external review requirements or achieving impact targets’; Wöckener et al. (2018): ‘Issuers are reluctant to include “green” undertakings as a breach may trigger an event of default under bond documentation, which could result in cross-defaults of other agreements’; Doran and Tanner (2019), p 23: ‘Failures to use the bond proceeds for stated green projects (or deliberate use for non-green purposes) and inadequate annual reporting (or simple noncompliance) are … not events of default or put events that would enable the noteholders to accelerate or redeem their bonds in the event of breach. Nor are they step-up events, which trigger an increase in the coupon payable by an issuer.’

62 Park (2018), p 13.

63 As noticed by Ehlers et al. (2020), p 34, even if projects financed by green bonds bring some environmental benefits, green bonds usually do not touch upon the question of the overall environmental performance at the firm level. It means, for example, that carbon emissions reductions achieved in the course of a project financed by green bonds could be offset by carbon increases of the same firm elsewhere.

64 Freeburn and Ramsay (2020), p 440.

65 Ibid.

66 Chan (2021), p 249.

67 See an example of such a contractual stipulation, provided by Doran and Tanner (2019), p 23: ‘Although the Issuer may agree at the Issue Date to allocate the net proceeds of the issue of the Green Bonds towards the financing and/or refinancing of Eligible Green Assets … it would not be an event of default under the Green Bonds if (i) the Issuer were to fail to comply with such obligations or were to fail to use the proceeds in the manner specified in this Offering Circular …’. Also Corke et al. (2019): ‘The historical market position is the inclusion, in the pricing supplement for an issuance programme, of an express statement that a loss of certification does not constitute an event of default and bondholders cannot exercise redemption rights or take any other action.’

68 Chan (2021), p 249: ‘…the issuance of green bonds has been observed to mostly mirror conventional bonds in the sense that there are no or very few modified provisions to accommodate the fact that they are thematically green …. As long as the interest and principal are paid, the bondholder often has no recourse.’

69 Ibid., p 251: ‘Perhaps the most cynical view of all is that investors simply do not have the time or resources to critically analyse the detailed terms of each bond, and therefore, do not care. Bondholders do not have the resources to initiate and process a bond restructuring by way of a bondholder meeting and would rather exit by a sale of the bonds.’

70 Ibid., p 249.

71 Doran and Tanner (2019), p 24.

72 On the reputational motivations of issuers, see ibid., p 23.

73 Chan (2021), p 250. As remarked by McGarry et al. (2018), p 179, in the absence of contractual stipulations on green default, bondholders sometimes rely on the tort of misrepresentation and other sanctions applicable to mainstream vanilla bonds.

74 The notion of ‘EU “green” label’ means that a given bond has the ‘green’ characteristic, attached to this bond under the European Green Bond Standard. See Badenhoop (2022), p 11. The European Green Bond Standard, similarly to private standards for the issuance of green bonds, will function as a ‘labelling’ standard. Green bonds issued under private standards for the issuance of green bonds are often referred to as ‘labelled bonds’. See Maltais and Nykvist (2020), pp 1 and 5.

75 European Central Bank (2021), pt. 3.2.2.

76 European Parliament Committee on Economic and Monetary Affairs (2022).

77 Freeburn and Ramsay (2020), p 441. Also, Wilkinson et al. (2021).

78 Recitals 1 and 2 to the Proposal for a Regulation on European green bonds.

79 Doran and Tanner (2019), p 25.

80 McGarry et al. (2018), p 179: ‘[I]f a green loan borrower fails to use the proceeds for green purposes, this can cause an event of default or trigger built-in penalties until the borrower cures it. In other market examples, if the quantitative sustainability targets set in a facility agreement are not met, a higher interest rate can be charged on a green loan.’ Also, Freeburn and Ramsay (2020), p 441.

81 On divergent national approaches to the question of civil liability for infringements of capital market rules: Veil (2010), pp 417–420.

82 Doran and Tanner (2019), p 25.

83 See n. 57.

84 European Commission (2021b), pp 6–7.

85 On GSS bonds: Giráldez and Fontana (2022), pp 10–13.

86 European Parliament Committee on Economic and Monetary Affairs (2022), p 108.

87 Park (2018), p 36.

88 Ibid. According to International Capital Market Association (2021), amendments to the proposed EU Regulation would result in a contraction of the EU green bond market. Also, Rust (2022).

89 CICERO Shades of Green AS (2022). See also Ehlers and Packer (2016), p 4. On various forms of green bond certification, see Ehlers T, Packer F (2017), pp 92–96.

90 The establishment of the EU Green Bond Standard was announced in the European Green Deal Investment Plan: European Commission (2020a).

91 European Commission (2020c).

92 European Central Bank (2021), pt. 1.5.

93 Ehlers et al. (2020), p 33, who notice that while the EU Green Bond Standard is envisaged to be voluntary once it is adopted, it is likely to become a de facto requirement for issuers in the European market.

94 European Central Bank (2021), pt. 3.1.2: ‘[T]he ECB considers it important that the EuGB becomes the prime green bond standard within the Union. Therefore, a clear commitment to making the standard mandatory for newly issued green bonds within a reasonable time period, while outstanding green bonds would maintain their designation as green bonds for a longer time period, is considered necessary.’

95 Within a hybrid regulatory framework, private governance regimes work parallel to or in cooperation with government regulatory bodies: Park (2018), p 38.

96 The answer to this question will depend on the final scope of the EU Taxonomy. If the EU Taxonomy were restrictively shaped, the EU Green Bond Standard could have an aggravating effect on the EU green bond market. See Freeburn and Ramsay (2020), p 434: ‘The development of green bond standards does not inevitably encourage green bond issuance. For example, it is estimated that under the EU’s proposed taxonomy, potentially 70 per cent of current issuance outstanding would not be compliant, with the effect of this being to restrict issuance, rather than encouraging it by providing clear definitions.’

97 EU Platform on Sustainable Finance (2022).
